# Paradigm Shift: A Comprehensive Review of Ovarian Cancer Management in an Era of Advancements

**DOI:** 10.3390/ijms25031845

**Published:** 2024-02-03

**Authors:** Valéria Tavares, Inês Soares Marques, Inês Guerra de Melo, Joana Assis, Deolinda Pereira, Rui Medeiros

**Affiliations:** 1 Molecular Oncology and Viral Pathology Group, Research Center of IPO Porto (CI-IPOP), Pathology and Laboratory Medicine Department, Clinical Pathology SV/RISE@CI-IPOP (Health Research Network), Portuguese Oncology Institute of Porto (IPO Porto), Porto Comprehensive Cancer Centre (Porto.CCC), 4200-072 Porto, Portugal; valeria.tavares@ipoporto.min-saude.pt (V.T.); ines.soares@ipoporto.min-saude.pt (I.S.M.); ines.melo@ipoporto.min-saude.pt (I.G.d.M.); 2 Faculty of Medicine, University of Porto, 4200-072 Porto, Portugal; 3 ICBAS—Instituto de Ciências Biomédicas Abel Salazar, University of Porto, 4050-313 Porto, Portugal; 4 Faculty of Sciences, University of Porto, 4169-007 Porto, Portugal; 5 Clinical Research Unit, Research Center of IPO Porto (CI-IPOP), RISE@CI-IPOP (Health Research Network), Portuguese Oncology Institute of Porto (IPO Porto), Porto Comprehensive Cancer Center (Porto.CCC), 4200-072 Porto, Portugal; joana.assis@iporto.min-saude.pt; 6 Oncology Department, Portuguese Institute of Oncology of Porto (IPOP), 4200-072 Porto, Portugal; dpereira@ipoporto.min-saude.pt; 7 Faculty of Health Sciences, Fernando Pessoa University, 4200-150 Porto, Portugal; 8 Research Department, Portuguese League Against Cancer (NRNorte), 4200-172 Porto, Portugal

**Keywords:** ovarian neoplasms, biomarkers, early detection of cancer, diagnosis, antineoplastic protocols

## Abstract

Ovarian cancer (OC) is the female genital malignancy with the highest lethality. Patients present a poor prognosis mainly due to the late clinical presentation allied with the common acquisition of chemoresistance and a high rate of tumour recurrence. Effective screening, accurate diagnosis, and personalised multidisciplinary treatments are crucial for improving patients’ survival and quality of life. This comprehensive narrative review aims to describe the current knowledge on the aetiology, prevention, diagnosis, and treatment of OC, highlighting the latest significant advancements and future directions. Traditionally, OC treatment involves the combination of cytoreductive surgery and platinum-based chemotherapy. Although more therapeutical approaches have been developed, the lack of established predictive biomarkers to guide disease management has led to only marginal improvements in progression-free survival (PFS) while patients face an increasing level of toxicity. Fortunately, because of a better overall understanding of ovarian tumourigenesis and advancements in the disease’s (epi)genetic and molecular profiling, a paradigm shift has emerged with the identification of new disease biomarkers and the proposal of targeted therapeutic approaches to postpone disease recurrence and decrease side effects, while increasing patients’ survival. Despite this progress, several challenges in disease management, including disease heterogeneity and drug resistance, still need to be overcome.

## 1. Introduction

In 2020, ovarian cancer (OC) was the eighth most diagnosed malignancy worldwide, affecting approximately 314,000 women and also ranking as the eighth most deadly cancer, with over 207,000 attributed deaths [1,2]. Like other tumours, the incidence and mortality of OC vary worldwide. While the disease is more common in European countries with high Human Development Index (HDI) levels, the lowest incidence rates are observed in African countries with a low HDI. In opposition, the mortality rates tend to have a reversed inclination [2,3].

Worldwide, OC has consistently been regarded as the most lethal gynaecological tumour. Despite improvements in disease management, particularly in surgical techniques and maintenance therapy (treatment after the first-line therapeutic approach to delay disease recurrence), OC patients still have a 5-year survival rate lower than 50% in most countries [4]. This is primarily driven by late disease diagnosis, owing to its non-specific symptoms and the lack of appropriate screening methods, combined with the frequent acquisition of chemoresistance leading to disease recurrence [5,6].

**Figure 1:**
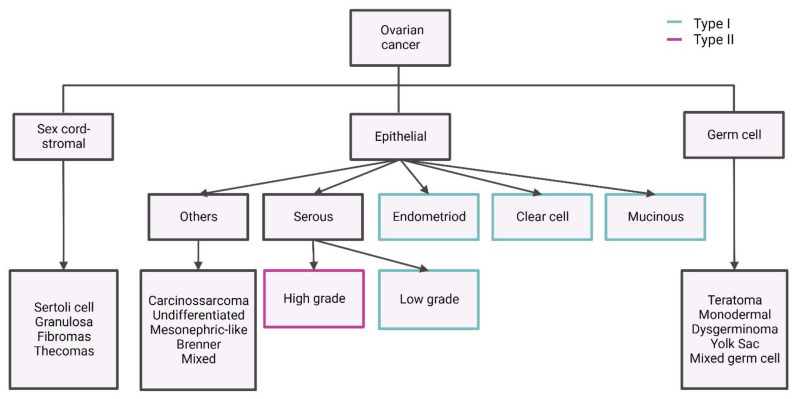
Subtypes of ovarian cancer. Figure created with BioRender.com (accessed on 28 December 2023). Ovarian cancer is a heterogeneous disease, encompassing numerous malignant subtypes with distinct aetiology, origin, pathogenesis, differentiation, spread patterns, and molecular profiles. The most common subtype is epithelial ovarian cancer (~90%), which can be further subclassified into type I and type II according to specific histological and molecular features [10,11,12,14,15].

Although ovarian tumourigenesis is poorly comprehended, the disease is thought to arise from the ovarian surface epithelium. Also, it is closely related to tumours originating from the peritoneum and the fallopian tube, according to the serous tubal intraepithelial carcinoma (STIC) theory [7]. Indeed, the three primary tumours are typically deemed as a single tumour entity classified as “ovarian or tubal cancers” [8,9]. Ovarian tumours constitute a heterogeneous group of malignant diseases with distinct aetiology, origin, pathogenesis, differentiation, patterns of spread, and molecular profiles [10]. According to the 2020 World Health Organization (WHO) classification, OC includes epithelial (EOC; 90%), germ cell (5%), and sex cord–stromal tumours (2–5%). EOCs (i.e., ovarian carcinomas) are the most common OC type, encompassing five main subtypes that are distinguished based on molecular analysis, histologic and immune profile: high-grade serous (HGSC; 70%), endometrioid (EC; 10%), clear cell (CCC; 10%), low-grade serous (LGSC; 5%) and mucinous (MC; 3%) carcinomas (Figure 1) [11,12]. According to the dualistic carcinogenesis model, these subtypes can be further subdivided into type I and type II according to specific histological and molecular features [13,14,15]. Type I tumours (~25% of EOCs) typically exhibit slow growth and tend to be diagnosed at earlier stages (stages I/II). Furthermore, these tumours appear to be associated with endometriosis and usually present a genetic stability phenotype with a pattern of mutations in *BRAF*, *KRAS*, *PTEN*, *CTNNB1*, *ARID1A*, *PIK3CA,* and *PPP2R1A*. Type II tumours (75% of EOCs), on the other hand, generally have rapid growth, with the disease being diagnosed at advanced stages (stages III/IV). These tumours also display a high degree of genetic instability, frequently exhibiting *BRCA* and *TP53* mutations. Despite the recognised clinical value of this classification system, it does not always reflect tumour aggressiveness, as even type I tumours can be very aggressive [14,15,16,17,18]. Not surprisingly, this heterogeneity impacts treatment response and clinical outcomes [10].

A paradigm shift has been observed in OC research with the evolution to a better disease understanding, aiming for effective screening, early diagnosis, and personalised treatment strategies. This shift was catalysed by innovations in genomics, including the widespread use of microarrays and next-generation sequencing (NGS), which have enabled cost-effective germline and tumour genomic profiling [19]. Notably, the available technology has led to the identification of more disease subtypes related to the molecular and genetic makeup of ovarian tumours (see Section 3) [20]. Furthermore, progress in molecular pathology, particularly integrating artificial intelligence and machine learning technologies, is shown to be determined [21]. Not dismissing technical and ethical challenges, existing data advocate that artificial intelligence models may aid in early and accurate OC diagnosis while providing important prognostic information to guide disease treatment [22].

Given the recent advancements in OC management, an in-depth overview of the current knowledge is critical for researchers and healthcare professionals to stay updated with the latest developments. Furthermore, it could help pinpoint research gaps and guide future investigations. Therefore, this comprehensive narrative review article aims to discuss the current body of evidence on the aetiology, prevention, diagnosis, and treatment of OC, highlighting recent progress in disease management and future directions for OC research. To perform this, a search in the PubMed database was conducted using combinations of the terms “ovarian cancer”, “ovarian tumour”, “ovarian carcinoma”, “advances”, “updates”, “overview”, “screening”, “prevention”, “diagnosis”, “prognosis”, “therapy” and “treatment” that appeared anywhere in the article. The retrieved papers were published between 2013 and 2023. Additional relevant publications were identified in the references list of the retrieved papers.

## 2. Disease Aetiology and Prevention

Various aetiological determinants are thought to impact ovarian tumourigenesis showing heterogeneity depending on tumour histology [3,23,24]. The most impactful ones are advanced age, genetic predisposition, and a family history of cancer. These factors are particularly related to continuous ovulation, hormonal changes, cumulative genetic damage, and chronic inflammation [3,25,26,27]. Ovarian tumours are rare among young women, particularly those under the age of 30. After the age of 50, especially following menopause, OC risk drastically increases, with the average diagnosis occurring between 50 and 70 years [12]. Concerning the genetic component, OC is one of the most heritable tumours, mainly linked to germline genetic mutations associated with the hereditary breast and OC syndrome (predominantly mutations in *BRCA1* and *BRCA2*) and hereditary nonpolyposis colorectal cancer syndrome (mutations in *MLH1*, *MSH2*, *MSH6,* and *PMS2*) [25,28]. Thus, a family history of breast, ovarian, and colorectal tumours, particularly at young ages, could be indicative of a high risk of OC onset [29,30]. For instance, while the risk of developing OC in the general population is <2%, women with *BRCA1* and *BRCA2* mutations have an overall lifetime risk of 20–40% and 10–20%, respectively [31].

Despite inconsistent data, reproductive factors such as early menarche, late menopause onset, long-term hormone replacement therapy, and nulliparity also constitute risk factors [32,33,34,35,36]. In opposition, pregnancy, breastfeeding, and the use of oral contraceptives are considered to be protective factors [37,38,39]. The impact of these determinants on a predisposition for OC is commonly attributed to the cumulative number of ovulatory cycles, as fewer cycles are associated with a lower OC risk [12,40,41]. Also, oestrogen exposure could be a contributing factor [42,43]. Other important risk determinants include lifestyle-related factors (e.g., diet, tobacco use, high body mass index, and obesity), a history of gynaecological conditions (e.g., endometriosis, ovarian cysts, and pelvic inflammatory disease), a personal history of endometrial, breast or colorectal cancers and ethnicity [44,45,46,47].

Identifying predisposing factors for OC development is important for tailoring prevention measures. However, there is no effective method for OC’s primary prevention. Nonetheless, tubal sterilisation and salpingo-oophorectomy for women at high risk, particularly those with hereditary syndromes, are possible prophylactic options. As such, according to the National Comprehensive Cancer Network (NCCN) guidelines (version 2.2021, 2021), genetic testing should be offered to women with a family history of the disease [48,49,50]. Furthermore, although conflicting, some studies have found that low-dose aspirin and other anti-inflammatory medications may decrease the risk of OC [40,51,52,53].

The secondary prevention of OC, which refers to disease screening, has also been challenging [54,55]. Ideally, an adequate screening exam should be easy to conduct, steadily reliable, inexpensive, and induce minimal discomfort. Importantly, it must have high sensitivity and specificity. For instance, an adequate test to screen for OC should have a sensitivity and specificity superior to 75% and 99.6%, respectively, to reach a positive predictive value (PPV) of 10% [56]. Also, a suitable exam should target the subpopulation with the highest prevalence of this condition of interest to establish an adequate PPV. Lastly, it should improve the morbimortality rates in the target population [54,57]. Several potential methods for OC screening have been reviewed, including serum CA-125 measurement, a transvaginal ultrasound, colour Doppler ultrasonography, and pelvic examination. However, none of them have shown adequate performance in trials involving the general population and high-risk groups [57,58,59,60]. For instance, CA-125 (also known as mucin 16 or MUC16), which is widely used in the clinical setting for OC monitoring, exhibits limited sensitivity in early disease stages. Also, its levels can be elevated in benign conditions such as ovarian cysts and endometriosis [24,61,62,63]. More recently, novel molecular markers have been proposed, including HE4, CA 72-4, CA 19-9, folate receptor alpha (FRα), microRNA profiles, DNA methylation patterns, circulating tumour DNA and antibodies in liquid biopsies, particularly blood and cervical mucus and swabs [62,64,65,66,67,68]. The use of liquid biopsies in disease screening is attractive since they can capture the disease’s heterogeneity through minimally invasive sample collection and at a low cost. However, the tumour material in these biopsies is usually scarce and does not provide information about the tumour’s architecture or its primary site [68]. According to existing data, a multimodal approach combining several tests might be the most effective tool to screen OC accurately [69]. In this context, several multivariate index assays have been proposed to help detect early-stage OC, including the risk of malignancy index (RMI), OVA1, and risk of ovarian malignancy algorithm (ROMA) [70,71,72,73]. Another advancement in this field is the development of new imaging techniques, namely auto-fluorescence and magnetic relaxometry, which could help detect the disease at earlier stages, enabling timely therapeutic intervention and better outcomes [67]. Despite these improvements, screening for asymptomatic and average-risk women is still controversial, given the low prevalence of this disease and the high probability of false-positive findings, which may lead to excessive interventions [74,75]. Consequently, 60–70% of OC patients are diagnosed at advanced stages upon symptom presentation, which, as formerly mentioned, significantly impacts their prognosis [5,12,76]. Of note, the list of possible symptoms encompasses vaginal bleeding, diarrhoea, constipation, abdominal distension allied to pain, eating difficulties, urinary frequency, fatigue, nausea, anorexia, dyspepsia, and early satiety [24]. The time of presentation of these symptoms may vary depending on the histological nature of the disease [77].

Given their implications, education on the risk factors underlying OC onset is crucial to increase patients’ health awareness and self-advocacy.

## 3. Disease Diagnosis and Prognosis Assessment

Current strategies to diagnose OC include a medical history evaluation combined with the gynaecological exam, serum CA-125 quantification, and imaging tests (transvaginal ultrasonography, computed tomography (CT), magnetic resonance imaging (MRI), and/or positron emission tomography (PET)), while also demanding a histopathological examination from either a diagnostic biopsy or, if possible, a surgical specimen for a definitive diagnosis and staging [78,79,80]. For MC, the evaluation of the tumour markers CEA and CA 19-9 is also recommended according to the European Society for Medical Oncology (ESMO) 2023 guidelines for OC management [24].

**Figure 2:**
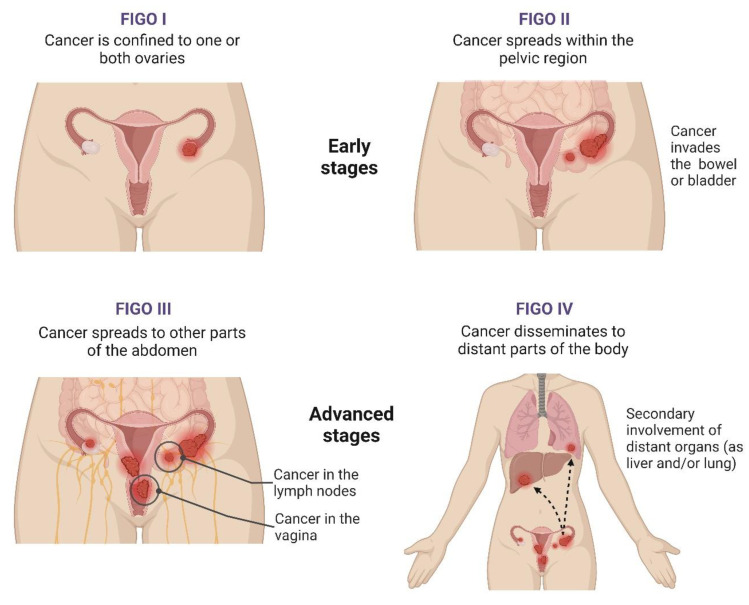
Stages of ovarian cancer. Figure created with BioRender.com (accessed on 28 December 2023). The FIGO staging system for ovarian cancer (OC) includes four stages that address the disease’s extent and severity by evaluating the tumour burden, dissemination within the abdomen, and the secondary involvement of distant organs. Stage I integrates tumours confined to either the ovary (one or both ovaries) or the fallopian tubes, while, at stage II, the tumour has already spread beyond the ovaries or fallopian tubes, with pelvic extension or primary peritoneal cancer. In stage III, OC cells spread to the peritoneum outside the pelvis, and there might be metastasis to the retroperitoneal lymph nodes. Lastly, stage IV is characterised by OC’s dissemination to other body parts beyond the pelvis and abdomen, namely the liver and lungs [82]. Abbreviations: FIGO, International Federation of Gynecology and Obstetrics.

At diagnosis, the International Federation of Gynecology and Obstetrics (FIGO) staging system is one of the most important tools to predict the clinical outcomes of OC patients and evaluate their therapeutical options [81]. This system, first published in 1973 and last revised in 2021, includes four stages, each with subdivisions (Figure 2) [75,81,82]. Ovarian carcinomas can also be subclassified based on histologic grading, with two systems being applied [60]. For non-serous tumours, according to cell architecture, the disease can be deemed as GX (grade not determined), G1 (well differentiated), G2 (moderately differentiated), and G3 (poorly differentiated). On the other hand, serous carcinomas can be categorised as low or high-grade based on their distinct cellular characteristics and behaviours [60,82].

Regarding the prognosis assessment, the FIGO stage, histologic subtype, grade, baseline serum CA-125 levels, the extent of debulking surgery, and chemotherapy schemes are traditionally deemed the most relevant independent prognostic factors of OC. For instance, those with early disease stages, type I tumours and lower baseline CA-125 levels usually have higher survival [12,83,84,85,86,87,88]. However, ongoing research has recently identified several molecular biomarkers associated with OC treatment response and prognosis, including mutations, gene expression patterns, and/or epigenetic changes [89,90,91]. This is particularly relevant given the high heterogeneity that characterises HGSC, with the predominant and most lethal OC subtype accounting for 70% of OC-related deaths [92]. Notably, Tothill et al., (2008) [93] were the first to propose HGSC subtypes based on the following genomic signatures: C1 (high stromal response), C2 (high immune signature), C4 (low stromal response) and C5 (mesenchymal). Next, Kurman and Shih (2010) [13] proposed the classic dualist model—type I vs. type II. Later, in 2011, data on histological structure and gene expression profile from the Cancer Genome Atlas (TCGA) Research Network led to the recognition of four HGSC subtypes: mesenchymal (with a gene expression profile that resembles mesenchymal tissues with increased cell motility and invasiveness), proliferative (displaying a molecular pattern indicative of high cell proliferation and limited inflammatory infiltration), differentiated (with a gene expression profile related to more specialised cell types) and immunoreactive (tumours with high infiltration of immune cells and with a gene expression profile characteristic of immune activation) [15,94]. Although not mutually exclusive, these subgroups correlate with prognosis. According to the “Classification of Ovarian Cancer” (CLOVAR) signature, the mesenchymal subtype is the most lethal with a related five-year OS of 18%, followed by the proliferative, differentiated, and, finally, the immunoreactive subtype, which is associated with a survival rate of 45% [95]. Importantly, these signatures also influence therapy response [96]. Since the proposal of these models, the integrative analysis of tumour (epi)genetic and molecular signatures has more or less confirmed the existence of these four HGSC subtypes with an impact on prognosis and/or treatment response (Table 1). This is anticipated to change OC management by facilitating personalised treatment [91].

## 4. Current Treatments and Innovations

The therapeutic management of OC mainly relies on the disease stage, with tumour histology, molecular profile, and the patient’s medical background also being relevant determinants. Traditionally, the front-line approach involves cytoreductive surgery followed by intravenous chemotherapy with platinum-containing drugs (cisplatin or carboplatin) typically combined with taxane agents (paclitaxel and docetaxel) every 21 days for six cycles [79,103,104,105,106]. According to ESMO 2023 guidelines, for patients at stage I and with low-grade tumours, chemotherapy can be omitted [24]. As for those with advanced disease, the complete resection of macroscopic disease (i.e., complete debulking) is often not conceivable. As such, these patients might first be treated with neoadjuvant (induction) chemotherapy, and if there is a treatment response, an interval debulking resection can be conducted, followed by adjuvant chemotherapy [75,107]. Radiotherapy is also a possible therapeutic approach; however, due to its high toxicity and low effectiveness compared to platinum-based chemotherapy, its use is often limited to palliative care [108,109,110].

Although the majority of OC patients (~80%) have a complete response after front-line treatment, over 60% of the patients with <1 cm of residual disease (optimal debulking) and about 80% of those with >1 cm of residual disease (suboptimal debulking) progress to around 18 months, often due to chemoresistance [12,105,111,112,113,114]. At a phase of disease recurrence, OC treatment commonly consists of second-line chemotherapy, which depends on platinum sensitivity [114]. Based on the period between the completion of first-line platinum-based chemotherapy and disease recurrence (i.e., platinum-free interval (PFI)), OC can be classified as platinum-refractory (when it occurs during the first-line chemotherapy), resistant (within 6 months after treatment completion), partially sensitive (between 6 and 12 months) or highly sensitive (beyond 12 months) [104,114,115]. According to ESMO 2023 guidelines for recurrent OC management, patients with sensitive disease can benefit from second-line chemotherapy with a combination of platinum compounds with paclitaxel, gemcitabine or pegylated liposomal doxorubicin (PLD), followed by treatment with bevacizumab (see Section 4.1) or poly (ADP-ribose) polymerase (PARP) inhibitors (PARPi) (see Section 4.2). In the event of platinum-hypersensitivity reaction/intolerance, PLD might be combined with trabectedin [24]. As for those refractory or resistant to platinum, the best therapeutical option is monotherapy with paclitaxel, gemcitabine, PLD, or topotecan, although the overall response rate with these agents is relatively small (8 to 20%) [24,114,116]. In this setting, bevacizumab can also be added if not contraindicated [24]. It is worth mentioning that most OC patients with recurrent disease eventually develop platinum resistance [117].

**Figure 3:**
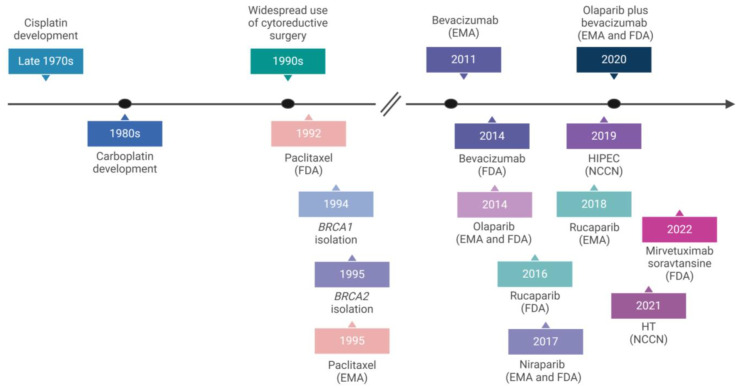
Pivotal gene discoveries and the approval of therapeutic agents and approaches to treat ovarian cancer. Figure created with BioRender.com (accessed on 28 December 2023). Several therapeutical agents and approaches for ovarian cancer management have emerged in recent decades due to a better understanding of the disease’s pathogenesis [122,123]. Abbreviations: EMA, European Medicine Agency; FDA, Food and Drug Administration; HIPEC, hyperthermic intraperitoneal chemotherapy; HT, hormonal therapy; NCCN, National Comprehensive Cancer Network.

The disease heterogenicity complicates OC treatment. The acquisition of chemoresistance can arise due to tumour microenvironmental, cancer cell-specific, and pharmacokinetic aberrations [116]. Additionally, chemotherapy is associated with adverse events, including but not limited to alopecia, neuropathy, neutropenia, palmar-plantar erythrodysesthesia, ototoxicity, and bone marrow depression, all of which negatively impact the patient’s quality of life [118,119,120,121]. Consequently, over the past few decades, a framework change has been observed, transitioning from an era of first-line treatment mainly centred around cytoreductive surgery followed by platinum and taxane-based chemotherapy to a new phase with improved upfront interventions, such as hyperthermic intraperitoneal chemotherapy (HIPEC), to delay the disease’s recurrence, reduce adverse effects and prolong patients’ survival. This evolution also encompasses broadening the treatment options to include more targeted approaches, namely the use of antiangiogenic agents, DNA damage repair-based therapeutics, hormone receptor modulators, and FRα-targeting drugs (Figure 3). These novel therapeutical agents target signalling pathways that are central to the progression of OC and/or its mechanism of drug resistance [87].

### 4.1. Antiangiogenic Agents

Tumours release proangiogenic factors, including VEGFA, which can activate the proliferation of vascular endothelial cells, fuelling tumour neoangiogenesis [124]. VEGFA and angiogenesis are crucial promoters of ovarian tumourigenesis. Both correlate directly with the disease’s extent and inversely with progression-free survival (PFS) and overall survival (OS), usually regardless of other prognostic determinants [125,126].

In 2011, after the results of the GOG-0218 (NCT00262847) and ICON7 (NCT00483782) trials, bevacizumab, a recombinant humanised anti-VEGFA monoclonal antibody, was approved by the European Medicine Agency (EMA) for the first-line and maintenance treatment of advanced-stage OC in combination with platinum-taxane-based chemotherapy [125,126]. Subsequently, in 2014, the US Food and Drug Administration (FDA) granted approval for this drug to be used in the second-line therapy of platinum-resistant-recurrent OC [127]. By neutralising all active forms of VEGFA, bevacizumab suppresses angiogenesis, inhibiting tumour growth and metastatic dissemination [128]. Additionally, it is thought to enhance the delivery of chemotherapeutic agents to their designated targets by normalising the tumour’s vasculature, decreasing the interstitial fluid pressure, and increasing the tumour’s oxygenation [129]. This agent was the first biological drug to show a promising therapeutic response in the frontline intervention (first-line therapy) and recurrent OC (second-line therapy) [125,130]. However, its effect on PFS is limited and does not prolong OS [131,132]. Also, bevacizumab is associated with considerable toxicity, with a list of adverse events including hypertension, thrombotic events, gastrointestinal perforation, and renal and central nervous system disorders [125,133,134].

There is no unanimous agreement on the prescription of bevacizumab, given the lack of validated predictive biomarkers of response [76]. Nevertheless, those with molecular subtypes associated with poor survival, namely proliferative and mesenchymal tumours, are known to benefit most from bevacizumab-based treatment [135]. More recently, its use in combination with PARPi has proven to be beneficial, receiving approval from both the EMA and the FDA in 2020 [123,136]. Furthermore, in addition to bevacizumab, small-molecule kinase inhibitors targeting VEGFA receptors (VEGFRs) are currently under investigation (see Section 5.4).

### 4.2. DNA Damage Repair-Based Therapeutics

Since 2014, the landscape of OC management has been revolutionised with the approval of PARPi by the EMA and FDA for disease treatment in different settings [137,138]. These therapeutic agents inhibit the activity of PARPs, which are proteins crucial for DNA damage repair. In malignancy, PARPs facilitate the repair of DNA damage, particularly single-strand breaks, which are induced by antineoplastic treatments [12,137]. Tumour cells with a deficient homologous recombination repair (HRR) pathway, mainly due to mutations in *BRCA1/2*, are unable to repair DNA double-strand breaks. In these cells, PARPi have a negative effect, rendering the repair of DNA damage unfeasible. Consequently, these therapeutic agents promote the apoptosis of tumour cells through a process known as synthetic lethality [12,137,139,140]. As anticipated, PARPi are particularly relevant for HGSC, given the high rate of HRR deficiencies [141].

For OC management, PARPi were initially proposed for patients with recurrent platinum-sensitive disease after the outstanding improvement in PFS observed in three randomised phase III trials—SOLO-2/ENGOT-OV21 (NCT01874353), NOVA/ENGOT-OV16 (NCT01847274) and ARIEL3 (NCT01968213) [137,142,143,144]. The results of these trials led to the approval of olaparib (2014), niraparib (2017), and rucaparib (2016–2018), respectively [137]. Early clinical data supported the effectiveness of these agents among those with germline or somatic *BRCA1/2* mutations. However, in the maintenance setting for those with platinum sensitivity, more recently, these drugs have shown clinical benefits even among those without these mutations [141]. Indeed, other genes implicated in the HRR pathway are known to be mutated in OC. The list includes *BARD1*, *BRIP1*, *RAD50*, *RAD51* paralogs (*RAD51C* and *RAD51D*), *MRE11* and *PALB2.* Curiously, OC patients present germline mutations in HRR-related genes more often than somatic tumour mutations (<10% of cases) [145]. In addition to recurrent disease, PARPi have been suggested to be beneficial in first-line therapy, which could affect subsequent treatment choices [137].

Despite these clinical benefits, the therapeutical impact of olaparib, niraparib, and rucaparib is constrained, translating into only a short-term survival extension as most patients inevitably develop drug resistance [146,147]. Therefore, other PARPi have emerged, including veliparib, pamiparib, fuzuloparib (formerly known as fluzoparib), and talazoparib. Veliparib is still under investigation, pamiparib and fuzuloparib were recently approved for OC treatment in China, and talazoparib was approved by the FDA in 2018 to manage HER2-negative-advanced breast cancer [148,149]. Moreover, the panorama of DNA damage repair-based therapies for OC management has evolved beyond PARPi with the development of pharmaceutical agents targeting the cell cycle checkpoint protein kinases ATR (ceralasertib), CHK1 (prexasertib) and WEE1 (adavosertib) [150]. These agents are still being investigated in clinical trials and promise to overcome PARPi-resistant ovarian tumours [150,151].

### 4.3. Hyperthermic Intraperitoneal Chemotherapy (HIPEC)

HIPEC involves the intraperitoneal delivery of chemotherapeutic agents after cytoreductive surgery and under hyperthermic conditions to improve patients’ outcomes by more effectively removing residual disease. This is partially due to hyperthermia, which increases the penetration of chemotherapeutic drugs at the peritoneal surface while enhancing the sensitivity of the tumour to treatment. These two factors, however, notably depend on the selected drug and the achieved temperature [152,153].

While HIPEC has been adopted in the management of malignant diseases such as colorectal, gastric, and primary peritoneal carcinomatosis, for OC, its implementation has been a subject of intense debate [154]. Only in 2019 was it integrated as an optional form of treatment for the interval debulking of OC patients in the NCCN guidelines (version 1.2019, 2019) [155]. In part, this delay was due to questions on optimal patient selection, the protocol for drug delivery (open versus closed), the timing of the treatment, the choice of drug regimen, and, importantly, the risk of complications [156]. Currently, according to the NCCN guidelines, HIPEC is recommended for OC patients with peritoneal carcinomatosis (FIGO stage III) and with response or stable disease after undergoing neoadjuvant chemotherapy [155]. For these patients, the treatment has been associated with a trend towards improved PFS and OS [157]. However, for various reasons, despite the demonstrated benefits, the acceptance and implementation of HIPEC by gynaecologic oncology and surgeons have been challenging [24,158,159].

### 4.4. Hormone Receptor Modulators

Oestrogen is known to drive the proliferation of OC cells [160]. Oestrogen signalling is mediated by oestrogen receptor(ER)-alpha (ERα) and ER-beta (ERβ), each with different isoforms, which are further amplified by G protein-coupled oestrogen receptor 1 (GPER1) [42]. In vitro and in vivo studies show that oestrogen via ERα regulates OC growth and promotes cell migration and epithelial–mesenchymal transition (EMT), influencing cell motility and survival [161,162,163,164]. These modifications proceed through the downregulation of E-cadherin: a process that ERβ inhibits [165]. Indeed, ERβ, the most common ER form in normal ovary tissue, is thought to be an OC suppressor [166,167,168,169]. As for GPER1, both suppressive and promotor roles have been proposed among OC patients, indicating a likely complex function [170,171,172,173,174]. Contrary to MC (21%) and CCC (20%), over 80% of serous EOCs (HGSC and LGSC) and EC express ERα and have demonstrated response to hormonal therapy with aromatase inhibitors (for instance, letrozole) and tamoxifen in multiple clinical studies [42,50,76,175,176]. While aromatase inhibitors block oestrogen synthesis, tamoxifen directly competes with oestrogen in order to bind to ER [177]. Progesterone, gonadotropins, androgens, and the gonadotropin-releasing hormone (GnRH) also play a role in the endocrine regulation of the ovary mediated by the hypothalamic–pituitary–ovary axis. While GnRH and progesterone seem protective against OC, gonadotropins, and androgens favour its progression [111,178].

The restricted therapeutic options for the management of recurrent and platinum-resistant OC and the favourable safety profile combined with its convenient and inexpensive use make hormonal therapy an attractive option [117]. According to the ESMO-European Society of Gynaecological Oncology guidelines (ESMO-ESGO) of 2019 and the NCCN guidelines (version 2.2021) of 2021, hormonal therapy is recommended as an alternative approach to treat those with recurrent and platinum-resistant OC [50,76]. However, the clinical benefit of hormonal therapy in OC management has not been systematically evaluated in large trials (arzoxifene, an ER modulator, in NCT00003670; fulvestrant, an ER degrader, in NCT00617188, tamoxifen in NCT02728622 and NCT00041080; and mifepristone, a progesterone receptor modulator, in NCT00459290 and NCT02046421). Currently, efforts are being made to identify biomarkers that can stratify responsive OC subgroups [42].

### 4.5. FRα-Targeting Drugs

The folate metabolism is essential in DNA synthesis, methylation, and repair [179]. The transmembrane glycoprotein FRα transports folic acid (folate) and its derivatives into cells via endocytosis [180]. In normal tissues, its expression is restricted to the intestine, kidney, retina, lung, choroid plexus, and placenta [181]. Except for the kidney (which does not retain folate), FRα in normal tissues is only presented in polarised epithelial cells, which are inaccessible to circulating pharmaceutical agents [181,182]. On the other hand, its elevated expression is demonstrated in most carcinomas, including endometrial, breast, lung, and ovarian tumours. This selective expression and its ability to be internalised after ligand-binding makes FRα an attractive target for cancer drug delivery [179].

Most ovarian carcinomas overexpress FRα, while this receptor is absent in normal ovarian epithelium [182,183]. The synthesis of FRα is particularly common in advanced and high-grade serous EOC, which is sustained even in recurrent diseases and within metastatic niches [184]. Importantly, this receptor is reported to shed from the cell membrane into circulation [66]. In EOC patients, circulating receptor (sFRα) levels correlate with tumour FRα expression, disease burden, and treatment outcomes [66,185]. Thus, sFRα might be an attractive biomarker of early EOC. Inclusively, this marker has exhibited higher accuracy than serum CA-125 levels [66].

In the treatment setting, FRα-targeting drugs have emerged as potential therapeutic agents for OC. Antibody-drug conjugates (ADCs) are a group of agents designed to selectively deliver chemotherapeutic agents to the site of tumours by targeting cancer-specific antigens [186]. Mirvetuximab soravtansine, one of the most extensively studied FRα-targeting ADCs, is composed of an anti-FRα antibody coupled to a potent tubulin-targeting agent named DM4. Mechanistically, the drug binds to FRα in EOC, delivering DM4 directly to the tumour cells, providing a positive balance between efficacy and toxicity. Currently, mirvetuximab soravtansine is being tested for EOC management in platinum resistance [186,187]. Based on the positive findings of the phase III trial SORAYA (NCT04296890), this drug received accelerated approval in 2022 by the FDA for the treatment of patients with FRα-positive and platinum-resistant EOC previously treated with systemic anticancer regimens [188]. Another FRα-based therapeutic strategy involves farletuzumab, a humanised monoclonal antibody to FRα. Particularly in low-folate environments, FRα provides a growth advantage to cancer cells. As expected, farletuzumab demonstrated growth-inhibitory effects on FRα-expressing OC cells in preclinical models [189,190]. Yet, clinical trials managing platinum-sensitive EOC with this drug in combination with other therapeutical approaches have shown conflicting results (NCT00318370 and NCT02289950) [191,192].

## 5. Emerging Therapies

The existing evidence indicates a stagnation in OC therapies, failing to extend the OS of patients significantly. As a result, there is a pressing demand for novel treatment approaches. Several therapeutical agents and schemes are being developed or are currently undergoing clinical trials, displacing encouraging preliminary results.

### 5.1. Immunomodulators

One of the emerging therapies for OC is cancer immunotherapy. This therapeutic method harnesses the power of the patient’s immune system to eliminate the tumour [193]. Numerous immune-based interventions have been approved to treat solid and haematologic tumours, including immune checkpoint inhibitors, nonspecific immune stimulation, adoptive cell therapy, and cancer vaccines [194]. The involvement of the immune system in OC patients’ outcomes is demonstrated by the observation that tumour-infiltrating lymphocytes and the lower expression of PD-L1 are associated with improved survival [195,196,197,198]. Considered an “inflamed tumour”, OC could benefit from these immune-based interventions, yet data are insufficient and inconsistent [199,200]. Thus, multiple clinical trials have explored the role of OC immunotherapy as a standalone treatment and in combination with other therapeutical approaches, namely chemotherapy, the use of antiangiogenic agents, and PARPi [199,200]. The studies actively recruiting are described in Table 2. Current studies, including (epi)genetic and molecular profiling, are also focused on identifying predictive biomarkers to assess the responsiveness of OC to immune-based interventions and improve patient selection criteria [199,201]. Namely, tumour mutational burden (TMB), meaning the number of somatic mutations per unit of a tumour-interrogated genome, has surfaced as an important marker of response to immune checkpoint inhibition [202]. In 2020, the FDA granted accelerated approval to pembrolizumab (anti-PD-1 agent) for the treatment of unresectable and/or disseminated solid tumours with high TMB (≥10 mut/Mb) [203]. The exploration of immunotherapy and the integration of predictive biomarkers in clinical decision-making represent promising strides in the personalised management of OC.

### 5.2. Gene Therapies

Gene therapy is generally defined as the replacement of an abnormal gene with a functional copy of that gene aiming to correct an underlying disorder [204]. Different gene therapy strategies have been explored for OC management in preclinical studies, including the replacement of tumour suppressor genes to restore cell control (e.g., *TP53*), oncogene inhibition strategies (e.g., *EGFR*), suicide gene therapy with the delivery of genes encoding for toxins (e.g., *HSV-TK*), genetic immunopotentiation to reinforce immune response against tumour cells (e.g., *IL-12A/B*), antiangiogenic gene therapy (e.g., *COL18A1*), strategies to restore pharmacological sensitivity (e.g., survivin (*BIRC5*)) and cancer virotherapy (e.g., vesicular stomatitis virus). Furthermore, some of these approaches have also been investigated in clinical trials (Table 3). Despite continuous progress and promising results, several challenges prevent the clinical implementation of gene therapy, including low efficiency in the delivery of therapeutic genes, an unspecific expression allied to biosafety concerns, and ethical and financial issues. In addition, OC, like other malignant diseases, is a polygenic disease characterised by a higher degree of heterogeneity between individuals and even tumours in the same patient [204,205,206]. Thus, more clinical trials are required to explore the current preclinical strategies and the correct way to translate gene therapy to the clinical setting.

### 5.3. Drug Repurposing

Drug repurposing (also known as drug reprofiling, re-tasking, or repositioning) consists of identifying alternative uses for approved therapeutical agents that are outside the original prescription scope, even regarding non-cytotoxic drugs [207]. This strategy cuts research costs and speeds up drug usage as the repurposed drugs have already been deemed safe in preclinical models and humans. As a result, drug repurposing has achieved great success, leading to the identification of candidate drugs for a pleura of diseases [208].

Focusing on the therapeutic agents approved for non-oncological diseases, one of the repurposed drugs under investigation for OC management is vitamin D (VD) and its analogues. VD consists of a group of steroid-like molecules, namely cholecalciferol (vitamin D3), ergocalciferol (vitamin D2), calcidiol (25-hydroxy-vitamin D) and calcitriol (with the active form also known as 1,25-dihydroxy vitamin D3 or 1,25D3), with the latter binding to the vitamin D receptor (VDR) to modulate the expression of several genes [209]. The most studied role of VD and its analogues is the maintenance of serum calcium and phosphorus homeostasis. Beyond their functions in physiological conditions, these steroid-like molecules are also reported to have antitumour effects in preclinical models. Namely, they can induce tumour cell differentiation and apoptosis while reducing the cells’ proliferation and dissemination potential [209,210,211]. Consequently, synthetic VD analogues, which do not possess the side effect of hypercalcemia, have been developed to target malignant diseases [212]. Many epidemiological studies have linked VD deficiency to cancer risk and mortality [213,214,215]. The implications of VD are best characterised by breast, colorectal, and prostate cancers [212]. Regarding OC, although in vitro and in vivo studies have obtained promising results, the impact of VD and its analogues is still blurred. Current evidence suggests that VD-based therapy could potentiate the activity of chemotherapeutic agents and PARPi [216,217,218,219,220,221]. The combination of VD with immunotherapy has also been considered potentially beneficial, given its immunomodulatory effect [222]. However, clinical trials assessing the efficacy of VD-based therapy in OC are lacking.

Other repurposed drugs have been investigated in clinical trials to help manage OC. This list includes statins (hypercholesterolemia; NCT04457089 and NCT00585052), hydroxychloroquine (malaria, rheumatoid arthritis and lupus erythematosus; NCT03081702), metformin (type 2 diabetes mellitus; NCT02312661 and NCT01579812), itraconazole (fungal infections; NCT03081702), beta-blockers (hypertension; NCT01504126) and sodium valproate (bipolar disorder and epilepsy; NCT00529022) [223,224]. Of note, the off-labelled use of drugs approved for other malignant diseases in OC management is beyond the scope of this review.

Given the implications of drug repurposing, more investigation in this field is needed to better understand the underlying mechanisms.

### 5.4. Small-Molecule Kinase Inhibitors

Kinases are implicated in several signalling pathways that are often deregulated in cancer. These proteins regulate cell survival and growth, promoting tumour progression [225]. In OC, the kinases involved in angiogenesis (e.g., VEGFRs), cell growth (e.g., EGFR), and intracellular signalling (e.g., PI3K/AKT/mTOR pathway) are reported to be overactivated, being attractive therapeutic targets [226]. Numerous small-molecule kinase inhibitors have been evaluated in clinical trials for OC management (Table 4). Despite their potential, the high heterogeneity of ovarian tumours and drug resistance are significant obstacles to the implementation of these drugs [226,227,228,229]. Nevertheless, progress in disease (epi)genetic and molecular profiling may help solve some of the current issues [91].

### 5.5. Coagulation-Targeting Approaches

Patients with ovarian tumours are commonly diagnosed with venous thromboembolism (VTE), with an incidence ranging from 10 to 30% [239]. This thrombotic event constitutes the second cause of death among oncological patients [240]. Importantly, even in the absence of VTE, most cancer patients present a state of blood hypercoagulation. Cumulative evidence suggests that underling this state, deregulated haemostatic components—endothelial cells, platelets, and coagulation/fibrinolysis systems—exhibit protumourigenic functions, including tumour cell growth, survival, proliferation, and invasion while also supporting cancer neoangiogenesis and metastatic dissemination [226]. Several haemostatic components have been suggested to play critical roles in OC progression and ascite formation, creating potential avenues for therapeutic intervention [241]. Namely, the overexpression of coagulation factor 3, commonly known as the tissue factor (TF), and the presence of tumour-educated platelets are some of the most studied mechanisms in this interface of VTE and OC progression [239,241,242].

Regarded as the initiator of the extrinsic coagulation pathway, TF is released into the blood circulation following vascular damage to trigger fibrin deposition at the injury site. This mechanism is vital to stop blood loss and restore haemostasis [243,244,245]. Cancer cells constitutively express TF and induce its synthesis in normal cells in the tumour microenvironment, which generates a prothrombotic cascade that favours tumour progression [246]. Inclusively, the overexpression of this coagulation factor in several tumour types, including OC, is linked to poor prognosis [247,248,249,250]. The protumourigenic roles of TF encompass tumour cell proliferation, cancer stemness, angiogenesis, immune evasion, and metastasis through clotting-dependent and independent processes [243,251,252,253]. Recently, tisotumab vedotin (Tivdak™), a TF-specific human ADC conjugated to the tubulin-targeting agent called monomethyl auristatin E (MMAE), was approved by the FDA for the management of recurrent or metastatic cervical tumour [254,255,256]. According to the results of phase I/II innovaTV-201 (NCT02001623), this drug showed promising antitumour activity and a convenient safety profile in platinum-resistant OC, which supports the continued investigation of tisotumab vedotin in this population [255].

Platelets, also known as thrombocytes, are megakaryocyte-derived haemostatic key players in the bloodstream [257,258,259]. Apart from their role in haemostasis, platelets promote tumour growth and dissemination, and, in turn, tumour cells stimulate platelet production and activation, creating a feedback loop that fuels tumourigenesis and leads to paraneoplastic thrombocytosis (i.e., an elevated platelet count >450,000 per cubic millimetre) [260,261]. This well-recognised phenomenon is often associated with many solid tumours [257,262]. In the context of OC, the interaction of thrombocytes and tumour cells is so evident that one-third of women with newly diagnosed OC have paraneoplastic thrombocytosis. However, data on the impact of thrombocytosis on the patient’s clinical outcomes is inconsistent [261,263,264]. Some studies have demonstrated that when considering other clinical factors, such as cancer burden, thrombocytosis in advanced OC-stage patients does not independently impact prognosis [261,265]. Others, nevertheless, have shown that this condition is associated with an advanced disease stage, high grade, and elevated preoperative CA-125 levels, which are all known OC prognostic factors [259,261]. When focusing on early stages, thrombocytosis seems to be a powerful prognostic factor, with affected patients exhibiting approximately an eightfold increase in the risk of recurrence and a fivefold increase in the risk of death. Moreover, this condition in these patients seems to correlate with disease burden, residual disease, and postoperative complications [265]. On the other hand, thrombocytosis was also found to be an independent prognostic factor, regardless of disease stage, tumour grade, histologic type, and the extent of surgical intervention (*p* < 0.001). Namely, affected patients presented a median OS of 2.65 years compared to the 4.65 years exhibited by their counterparts [261]. Thus, designing therapeutic agents to target platelets at the tumour microenvironment (i.e., tumour-educated platelets) can provide a promising breakthrough in OC treatment [266]. Epidemiological studies have suggested that acetylsalicylic acid (also known as aspirin), a non-steroidal anti-inflammatory drug, may have anticancer properties [267,268]. By inhibiting cyclooxygenase-2 (COX-2), aspirin exerts antiplatelet and anti-inflammatory effects. Although the cumulating data on aspirin’s impact on OC patients’ survival is conflicting, preclinical data show that aspirin exerts anti-tumoural effects when combined with bevacizumab [269,270]. Thus, the phase II trial EORTC-1508 (NCT02659384) is currently evaluating the efficacy and safety of combining atezolizumab (monoclonal antibody targeting PD-L1), bevacizumab and aspirin to treat recurrent platinum-resistant OC. Moreover, an ongoing phase I trial (NCT05080946) aims to evaluate the effectiveness of aspirin with neoadjuvant chemotherapy for decreasing markers of immune suppression (M2 tumour-associated macrophages and immunosuppressive T-regulatory cells) within the tumour. More studies on aspirin’s effect and the development of other antiplatelet agents for OC treatment should be evaluated.

## 6. Conclusions

Despite significant strides in disease management, OC remains the most lethal female reproductive cancer. Not dismissing the potential publication and selection bias, which is characteristic of narrative reviews, this comprehensive overview provides an in-depth analysis of the recent evidence regarding OC management, identifying gaps in the literature and suggesting future directions for disease research. Briefly, ongoing research focuses on dissecting OC pathogenesis to refine screening techniques and seek innovative and more targeted treatments to manage this malignant disease effectively, decrease side effects, and enhance OC patient outcomes. Due to a better understanding of OC’s (epi)genetic and molecular profiling, several therapeutical approaches have been recently approved, with others in development. Notably, (epi)genetic and molecular changes in ovarian tumours have been found to correlate with drug efficacy and resistance, particularly in HGSC heterogeneity. Thus, integrating molecular insights might have significant implications for clinical decision-making. Future investigation endeavours should be directed at OC’s heterogeneity and drug resistance challenges. Likewise, there should be an emphasis on identifying more accurate diagnostic tools, prognostic indicators, and predictive biomarkers of response for current and emerging therapies. Furthermore, more data should be collected on the impact of combining diverse treatment modalities, as it holds promise in elevating treatment effectiveness and conquering drug resistance, enhancing patient outcomes by leveraging the synergistic actions of multiple therapies.

## Figures and Tables

**Table 1:** (Epi)genetic and molecular signatures of high-grade serous ovarian carcinoma (HGSC) with implications for therapy response and patients’ clinical outcomes.

Authors (Year)	Number of Cases	Methods	HGSC Clusters and/or Main Features	Main Findings	References
Macintyre et al., (2018)	132 patients 112 patients (Pan-Cancer Analysis of Whole Genomes) 415 patients (TCGA)	Genome sequencing	Signatures of copy number variations: Signature 1—telomere shortening and RAS/MAPK activation; Signature 2—tandem duplication; Signature 3—BRCA1/2-related HRR deficiency; Signature 4—whole genome duplication; Signature 5—subclonal catastrophic chromothriptic-like events; Signature 6—focal amplification; Signature 7—non-BRCA1/2-related HRR deficiency.	Signature 1—platinum-resistant recurrence and poor survival; Signature 2—poor survival; Signatures 3 and 7—prolonged survival; Signatures 4, 5 and 6—unclear implications.	[97]
Harris et al., (2019)	Not reported	Tumour xenografting DNA and RNA NGS DNA fingerprinting Immunohistochemistry	DNA alternations in genes involved in the ERBB2 pathway.	Deregulation in the ERBB2 pathway—favourable results by combining platinum-based chemotherapy with anti-HER2 drugs.	[98]
Li et al., (2019)	Seven patients	Tumour xenografting RNA and whole exome sequencing Immunohistochemistry	Deregulation of *AKT3*, *HLA-DPA1*, *PIK3R5* and *SAP25* expression; *POLR2A* and *TMEM205* mutations.	Features associated with the acquisition of chemoresistance to carboplatin and paclitaxel.	[99]
McDonald et al., (2019)	450 patients with chemoresistance (TCGA)	Genome-wide cluster analysis Pathway enrichment analysis	Cluster 1—growth factor signalling; Cluster 2—cell survival; Cluster 3—cellular senescence.	Best therapeutic options: Cluster 1—tyrosine kinases or angiokinase inhibitors; Cluster 2—mTOR inhibitors; Cluster 3—deacetylase inhibitors.	[100]
Hao et al., (2021)	Two patients (four matched pair samples of primary and metastatic tumours)	Single-cell RNA sequencing	Cluster EC1—glycolysis/gluconeogenesis; Cluster EC2—cytokine–cytokine receptor interaction; Cluster EC3—nucleotide and amino acid metabolism; Cluster EC4—immune response Cluster EC5—DNA repair and drug metabolism	Cluster EC5 may be most aggressive and resistant to chemotherapy and PARP inhibitors.	[101]
Li et al., (2021)	66 tumour cells 568 tumour samples and 7 normal ovary samples (TCGA)	Single-cell RNA sequencing	Differently expressed genes	Low expression of *ANP32E*, *EGFL6*, *GPRC5A*, *PMP22* and *STAT1*—prolonged survival; Low expression of *ANP32E*, *CYB5R3* and *FBXO21*—prolonged PFS	[102]

Abbreviations: HRR, homologous recombination repair; NGS, next-generation sequencing; PFS, progression-free survival; TCGA, The Cancer Genome Atlas.

**Table 2:** Actively recruiting clinical trials of immunotherapy for OC management.

Clinical Trial	Trial Identifier	Immunotherapeutic Agent	Combination	N *	Phase	Setting	Reaction to Platinum	Completion Date *
Pembrolizumab and lenvatinib for the treatment of serous ovarian cancer patients	NCT05114421	pembrolizumab (anti-PD-1)	lenvatinib	30	II	First-line treatment Recurrent disease	NA NR	January 2024
Neoadjuvant dendritic cell vaccination for ovarian cancer (NEODOC)	NCT05773859	Specialised Cross-Presenting Dendritic Cells Vaccinations	Standard-of-care treatment	10	I/II	First-line treatment	NA	October2024
Systemic immune checkpoint blockade and intraperitoneal chemo-immunotherapy in recurrent ovarian cancer	NCT03734692	rintatolimod (immune system stimulant) pembrolizumab (anti-PD-1)	carboplatin	45	I/II	Recurrent disease	Sensitive	December 2024
Durvalumab and tremelimumab in treating participants with recurrent or refractory ovarian, primary peritoneal, or fallopian tube cancer	NCT03026062	durvalumab (anti-PDL1) tremelimumab (anti-CTLA-4)	-	120	II	Refractory diseaseRecurrent disease	Resistant	December 2024
Pembrolizumab and carboplatin for the treatment of recurrent ovarian, fallopian tube, or primary peritoneal cancer	NCT04387227	pembrolizumab (anti-PD-1)	carboplatin	22	II	Recurrent disease	NR	April 2025
PD-1 antibody combined neoadjuvant chemotherapy for ovarian cancer	NCT04815408	tislelizumab (anti-PD-1)	paclitaxel and carboplatin	40	II	First-line treatment	NA	April 2025
A clinical study on oncolytic virus injection (R130) for the treatment of relapsed/refractory ovarian cancer	NCT05801783	Recombinant oncolytic herpes simplex virus type 1 (R130)	-	10	I	Refractory diseaseRecurrent disease	NR	December 2025
OSE2101 alone or in combination with pembrolizumab vs. BSC in patient with platinum-sensitive-recurrent OC (TEDOVA)	NCT04713514	OSE2101 (cancer vaccine) pembrolizumab (anti-PD-1)	-	180	II	Recurrent disease	Sensitive	December 2025
Safety and efficacy of anti-CD47, ALX148 in combination with liposomal doxorubicin and pembrolizumab in recurrent platinum-resistant ovarian cancer	NCT05467670	pembrolizumab (anti-PD-1) ALX148 (anti-CD47)	PLD	31	II	Recurrent disease	Resistant	December 2027

Data available at “clinicaltrials.gov” (accessed on 29 December 2023) until December 2023 using the terms “ovarian cancer” and “immunotherapy” as keywords. Maintenance therapy was deemed a treatment strategy employed after the first-line therapy but preceding any disease recurrence. * Estimated. Abbreviations: NA, non-applicable; NR, non-restrictive; PLD, pegylated liposomal doxorubicin.

**Table 3:** Active and completed clinical trials of gene therapy for OC management.

Approach	Therapeutical Agent	Trial Identifier (Status)	Combination	N Participants	Phase	Setting	Reaction to Platinum
Replacement of tumour suppressor genes	Ad5CMV-p53 vector (Inserting *TP53*)	NCT00003588 (completed)	-	30	I	Recurrent disease	Resistant
		NCT00003450 (completed)	-	-	I	Recurrent disease	NR
Suicide gene therapy	Ad5.SSTR/TK.RGD vector (HSV-TK + GCV)	NCT00964756 (completed)	-	11	I	Recurrent disease	NR
Genetic immunopotentiation	p53MVA vaccine (Modified vaccinia virus Ankara expressing tumour protein p53)	NCT02275039 (completed)	gemcitabine	12	I	Recurrent disease	NR
	NYESO-1(C259)-transduced autologous T cells	**NCT01567891** (completed)	-	9	I/II	Refractory disease Recurrent disease	NR
	Vigil™ tumour cell vaccine	**NCT01309230** (completed)	-	145	II	First-line treatment	NA
	Gene-modified lymphocytes with MOv-PBL	NCT00019136 (completed)	aldesleukin	13–50 *	I	Residual disease Recurrent disease	NA NR
	CDX-1401 vaccine	NCT03206047 (active)	atezolizumab and guadecitabine	75 *	I/II	Recurrent disease	Resistant
	ALVAC(2)-NY-ESO-1 (M)/TRICOM vaccine	NCT01536054 (completed)	sirolimus and sargramostim	7	I	Maintenance therapy Recurrent disease	NA NR
		**NCT00803569** (completed)	sargramostim	13	I	Maintenance therapy Recurrent disease	NA NR
Cancer virotherapy	MV-CEA MV-NIS	**NCT00408590** (completed)	-	37	I	Refractory disease Recurrent disease	NR

Data available at “clinicaltrials.gov” (accessed on 29 December 2023) until December 2023 using the terms “ovarian cancer” and “gene therapy” as keywords. Maintenance therapy was deemed a treatment strategy employed after the first-line therapy but preceding any disease recurrence. Completed trials with results are highlighted in bold. * Estimated. Abbreviations: MOv-PBL, MOv-gamma chimeric receptor gene; MV-CEA, Carcinoembryonic antigen-expressing measles virus; MV-NIS, oncolytic measles virus encoding thyroidal sodium iodide symporter; NA, non-applicable; NR, non-restrictive.

**Table 4:** Active and completed clinical trials of small-molecule kinase inhibitors for OC management.

Inhibitor	Target	Trial Identifier (Status)	Combination	N Participants	Phase	Setting	Reaction to Platinum
anlotinib	VEGFRs, FGFRs, PDGFRs, c-Kit and RET kinases [230]	NCT05188781 (completed)	pembrolizumab	34	II	Refractory or recurrent disease	NR
		NCT05130515 (completed)	niraparib	6	II	Recurrent disease	Resistant
		NCT02584478 (active)	paclitaxel, PLD, topotecan and carboplatin	294	III	Recurrent or metastatic disease	NR
apatinib	VEGFR-2 [231]	NCT02867956 (completed)	etoposide	35	II	Refractory or recurrent disease	Resistant
		NCT03075462 (completed)	fuzuloparib	98	I	Recurrent	Sensitive
		NCT04348032 (active)	PLD	152	II	Recurrent disease	Resistant
		NCT04229615 (active)	fuzuloparib	690	III	Maintenance therapy	Sensitive
alpelisib	PI3K [232]	NCT04729387 (Active)	olaparib, paclitaxel and PLD	358	III	Refractory or recurrent disease	Resistant
cediranib	VEGFRs [233]	**NCT00275028** (completed)	-	47	II	Recurrent disease	Resistant
		**NCT00278343** (completed)	-	74	II	Refractory or recurrent disease	NR
		NCT02340611 (completed)	olaparib	4	II	Recurrent disease	NR
		**NCT02889900** (completed)	olaparib	62	II	Recurrent disease	Resistant
		NCT02681237 (completed)	olaparib	34	NA	Recurrent disease	NR
		NCT03117933 (active)	olaparib and paclitaxel	139	II	Recurrent disease	Resistant
		NCT02502266 (active)	olaparib, paclitaxel, PLD and topotecan	562	II/III	Recurrent disease	Resistant
		NCT02345265 (active)	olaparib	72	II	Recurrent disease	NR
		NCT02446600 (active)	olaparib	579	III	Recurrent disease	Sensitive
		NCT01116648 (active)	olaparib	155	I/II	Recurrent disease	Sensitive
erlotinib	EGFR [234]	NCT00217529 (completed)	docetaxel and carboplatin	30 *	I/II	First-line treatment	NA
		NCT00263822 (completed)	-	835	III	Maintenance therapy	NR
		NCT00030446 (completed)	carboplatin	50	II	Recurrent disease	NR
		NCT00126542 (completed)	bevacizumab	35	II	Recurrent or metastatic disease	NR
		**NCT00130520** (completed)	bevacizumab	40	II	Recurrent disease	Resistant
		**NCT00059787** (completed)	carboplatin and paclitaxel	56	II	First-line treatment	NA
		**NCT00520013** (completed)	bevacizumab, paclitaxel and carboplatin	60	II	Maintenance therapy	NR
palbociclib	CDK4 and CDK6 [235]	NCT01536743 (completed)	-	26	II	Recurrent disease	NR
pazopanib	VEGFRs, PDGFRs and FGFRs [236]	**NCT00281632** (completed)	-	35	II	Refractory disease	Refractory
		**NCT01227928** (completed)	-	145	II	Maintenance therapy	NA
		NCT01238770 (completed)	cyclophosphamide	10	I/II	Recurrent disease	Resistant
		NCT01644825 (completed)	paclitaxel	72	II	Refractory or recurrent disease	Resistant
		NCT01262014 (completed)	-	28	II	Recurrent disease	Resistant
		NCT01608009 (completed)	paclitaxel	16	I	Recurrent disease	Resistant
		**NCT00866697** (completed)	-	940	III	Maintenance therapy	NA
		**NCT01468909** (completed)	paclitaxel	106	II	Recurrent disease	Sensitive
		NCT01402271 (completed)	paclitaxel and carboplatin	88	I/II	Refractory or recurrent disease	Resistant
		**NCT01610206** (completed)	gemcitabine	148	II	Recurrent disease	NR
		NCT02383251 (completed)	paclitaxel	118	II	Refractory or recurrent disease	Resistant
sorafenib	Raf serine/threonine kinases, VEGFRs and PDGFR-β [237]	NCT00096395 (completed)	gemcitabine	33	II	Recurrent disease	NR
		NCT00093626 (completed)	-	73	II	Recurrent disease	Resistant
		**NCT00096200** (completed)	carboplatin and paclitaxel	44	II	Recurrent disease	Sensitive
		**NCT00791778** (completed)	-	246	II	Maintenance therapy	NA
		**NCT00390611** (completed)	paclitaxel and carboplatin	85	II	First-line treatment	NA
		**NCT00436215** (completed)	bevacizumab	55	II	Refractory or recurrent disease	Resistant
		NCT01047891 (completed)	topotecan	174	II	Recurrent disease	Resistant
sunitinib	VEGFR1, VEGFR2, PDGFR-α, PDGFR-β, c-Kit, FLT3, RET and CSF1R [238]	**NCT00388037** (completed)	-	31	II	Recurrent disease	NR
		**NCT00768144** (completed)	-	36	II	Refractory or recurrent disease	NR
		NCT01824615 (completed)	-	30	II	Recurrent disease	Resistant
		**NCT00979992** (completed)	-	35	II	Recurrent disease	NR

Data available at “clinicaltrials.gov” (accessed on 29 December 2023) until December 2023 using the terms “ovarian cancer” and “small-molecule kinase inhibitor” as keywords. Also, the term of each inhibitor was used. Maintenance therapy was deemed a treatment strategy employed after the first-line therapy but preceding any disease recurrence. Completed trials with results are highlighted in bold. * Estimated. Abbreviations: NA, non-applicable; NR, non-restrictive; OC, ovarian cancer; PLD, pegylated liposomal doxorubicin.

## Abbreviations

CCC: clear cell carcinoma; EC, endometrioid carcinoma; EMA, European Medicine Agency; EOC, epithelial ovarian cancer; ESMO, European Society for Medical Oncology; FDA, Food and Drug Administration; FRα, folate receptor alfa; HGSC, high-grade serous carcinoma; HIPEC, hyperthermic intraperitoneal chemotherapy; LGSC, high-grade serous carcinoma; MC, mucinous carcinoma; NCCN, National Comprehensive Cancer Network; OC, ovarian cancer; OS, overall survival; PARP, poly (ADP-ribose) polymerase; PARPi, Poly (ADP-ribose) polymerase inhibitors; PFS, progression-free survival.
